# My Broken Heart

**DOI:** 10.21980/J85W7R

**Published:** 2025-04-30

**Authors:** Kelly N Roszczynialski, Alana E Harp, Cameron A Fisk, Kristen M Ng, Ashley C Rider

**Affiliations:** * Stanford University, Department of Emergency Medicine, Palo Alto, CA; ^ Loma Linda University School of Medicine, Department of Emergency Medicine, Loma Linda, CA; † Weill Cornell Medical College, Department of Emergency Medicine, New York, NY

## Abstract

**Audience:**

The target audience for the key learning objectives of this Left-Ventricular Assist Device (LVAD) simulation are emergency medicine residents. Other team members such as attendings, nurses, pharmacists, and technicians could potentially be integrated.

**Introduction:**

Left ventricular assist devices (LVADs) are common bridge therapy for patients suffering from severe heart failure to cardiac transplant or destination therapy for non-transplant candidates.^1^ Emergency medicine physicians must be prepared for a variety of device complications that may result in an acute care presentation, such as drive-line infections, suction events, arrhythmias, and cardiac arrest with device failure. In a review investigating ED presentations for patients with LVADs, device-specific complaints were among the fewest, with the most common presentations involving bleeding, infection, and arrythmias.^2^ The present case involves a suction event that is precipitated by a gastrointestinal (GI) bleed, which has an incidence of 30% for LVAD patients.^3^ This case was developed for a technology failure-themed resident simulation competition during the Western Society for Academic Emergency Medicine (SEAM) conference held on April 1, 2022.

**Educational Objectives:**

By the end of this simulation session, learners will be able to: 1) assess the hemodynamics of an LVAD patient by using a Doppler to determine mean arterial pressure, 2) Manage an arrhythmia in an LVAD patient with a suction event by addressing preload, 3) Identify and treat the source of hypovolemia (a massive lower gastrointestinal hemorrhage), 4) Perform clear closed-loop communication with other team members.

**Educational Methods:**

This high-fidelity simulation case aims to train emergency medicine residents on recognition and management of an LVAD suction event, a rare but serious presentation encountered in the emergency department. This simulation can be successfully implemented either *in situ*, in an immersive simulation center, or off-site. This case could be represented by lower fidelity mannequins without the capabilities to provide learner tactile feedback of hemodynamics or airway, with a separate monitor device such as SimMon to display vital signs and digital media to demonstrate needed clinical images. The audio file of the low-flow alarm can be accessed and played by any device with internet access. The simulation benefits from embedded simulation participants to act as the bedside nurse and wife to provide history. This simulation included debriefing focused on a critical action checklist.

**Research Methods:**

A working group of two simulation-trained faculty, a simulation fellow, and three senior emergency medicine residents chose and developed the simulation case. Two simulation-trained faculty implemented the pilot case series to gather feedback on performance against the critical action checklist. One simulation-trained faculty then facilitated two additional *in situ* sessions, again evaluating performance on the critical actions as well as content of the debrief discussion. That data was used to iteratively edit the presentation and dynamics of the case in preparation for the SIMposium case competition.

**Results:**

During March 2022, in a three-case pilot *in situ* series, a total of 15 residents (five EM PGY4, four EM PGY3, five EM PGY2, one off-service PGY1) and two medical students (MS3) participated in the simulation case. Participant reactions were overwhelmingly positive, particularly from senior residents. The final version of the SIMposium case was held for a team of four emergency medicine residents from an alternate institution, all critical actions were met, and a discussion point arose regarding the reversal of anticoagulation in LVAD patients with acute GI bleed.

**Discussion:**

Overall, this simulation was well received, effective, and easy to implement and translate to immersive, *in situ*, or offsite locations for the training of emergency medicine residents on the management of a high acuity, low-frequency event of LVAD device complication. Each debrief stimulated an excellent discussion regarding the general management of LVAD patients regarding initial assessment, arrhythmia, and distinguishing pathologies from device alarms. Our main takeaway from this simulation was the power of a case involving a critical and high acuity patient with LVAD which stimulated residents to engage in more robust discussions during debriefing, leading to broader clinical learning.

**Topics:**

*In situ* simulation, simulation competition, LVAD, left ventricular assist device.

## USER GUIDE

**Table:** 

List of Resources:	
Abstract	1
User Guide	3
Instructor Materials	6
Operator Materials	20
Debriefing and Evaluation Pearls	23
Simulation Assessment	26

**Learner Audience:**

Interns, Junior EM Residents, Senior EM Residents, Ancillary Staff

**Time Required for Implementation:**

**Instructor Preparation:** Instructors should ensure they are comfortable with the topic of LVADs and LVAD complications. The instructor should plan to read background materials on the subject for one to two hours. The instructor should allow 30 minutes for mannequin and prop set up.

**Time for case:** The case should run for 15 minutes.

**Time for debriefing:** The allotted debrief time should be 15–25 minutes

**Recommended Number of Learners per Instructor:**

Number of learners should be one to four with at least one instructor.

**Topics:**

*In situ* simulation, simulation competition, LVAD, left ventricular assist device.

**Objectives:**

By the end of the session:

1. The learner will assess the hemodynamics of an LVAD patient by using a Doppler to determine mean arterial pressure (MAP).
2. The learner will manage an arrhythmia in an LVAD patient with a suction event by addressing preload.
3. The learner will identify and treat the source of hypovolemia as a massive lower gastrointestinal hemorrhage.
4. The learner will perform clear, closed-loop communication with other team members.
5. Develop a differential diagnosis for a critically ill patient with altered mental status
6. Discuss the management of myxedema coma in the ED, including treatments, appropriate consultation and disposition

### Linked objectives and methods

As with many simulation-based activities, we utilized Kolb’s Experimental Learning Cycle.^4^ Learners have the concrete experience of the 15-minute simulation, followed by an opportunity for reflection, and through the debrief, abstract conceptualization that can then be applied to the clinical setting. While learners in the initial concrete experience may not know how to successfully perform all of the objectives, after the debrief opportunity for conceptualization they will understand how to accomplish each objective.

Each objective was written according to Bloom’s Revised Taxonomy.^5^ The first three fall into the category of procedural knowledge within cognitive processes. The fourth falls into the affective domain.

### Learner responsible content

Learners do not need to perform any pre-reading; however, if the instructor chooses, the following Life in the Fast Lane blog offers a brief, easy-to-understand overview of LVAD types and complications:

- https://litfl.com/ventricular-assist-device-vad/

### Recommended pre-reading for instructor

Due to the complex nature of this topic, the facilitator should plan to spend a good deal of pre-work time reviewing the following references in order to be well-versed in LVAD physiology and complications.

- This paper is an excellent primer for emergency physicians and should be read in its entirety:
  ○ Sen A, Larson JS, Kashani KB, et al. Mechanical circulatory assist devices: a primer for critical care and emergency physicians. *Crit Care*. 2016;20:153. doi:10.1186/s13054-016-1328-z
- An article on managing GI bleeding in a patient with an LVAD:
  ○ Ahsan I, Faraz A, Mehmood A, Ullah W, Ghani AR. Clinical approach to manage gastrointestinal bleeding with a left ventricular assist device (LVAD). *Cureus*. 11(12):e6341. doi:10.7759/cureus.6341
- Management of critically ill LVAD patients:
  ○ Pratt AK, Shah NS, Boyce SW. Left ventricular assist device management in the ICU. *Crit Care Med*. 2014;42(1):158–168. doi:10.1097/01.ccm.0000435675.91305.76

### Results and tips for successful implementation

We implemented this case in two formats: first, a series of *in situ* simulation cases in the ED, and second, in a team-based simulation competition. The initial pilot case was in situ and tested on five residents (one EM PGY4, one EM PGY3, two EM PGY2, one off-service PGY1) and one medical student. A high-fidelity simulator, TraumaHal (Gaumard), was utilized as a low-fidelity mannequin with vital signs represented using SimMon for ease of set-up. The props initially used were a cranberry juice-soaked Chuck to simulate the lower GI bleed and a printout image of an LVAD HeartMate II showing a ‘Low Flow’ alarm message paired with the audio from open access YouTube video of LVAD alarms cued to appropriate Low Flow LVAD HeartMate II.

For the pilot case, two simulation-trained faculty were present, one acting as an embedded simulated nurse and the second managing the vital signs and presenting planned stimuli. The learners immediately recognized the low flow alarm and appropriately requested Doppler mean arterial pressure (MAP) and crystalloid. The learners eventually required cueing for the lower GI bleed from the simulated bedside nurse due to time constraints running an *in situ* sim during clinical environment. Upon seeing the ultrasound image of the collapsed left ventricle in a suction event, they interpreted the image as a dilated right ventricle, and paired with initial vital signs of hypoxia to 92%, began to pursue a diagnosis of pulmonary embolism. The case used a directed debrief of the following critical actions:

1. Using Doppler/MAP to assess perfusion in patient with an LVAD
2. Management of arrhythmia in patient with LVAD
3. Initial management with fluid resuscitation for low flow state in patients with LVAD
4. Differential for hypovolemia and suction event in patients with LVAD

Critical actions not completed during the simulation case served as teaching points during the debrief. The debriefings identified a learning point for each group regarding the tolerance of arrhythmia in patients with LVAD due to the mechanical pump maintaining perfusion. None of the resident teams were able to identify the suction event depicted in the bedside ultrasound image and interpreted it as an enlarged RV.

Two additional sessions were run *in situ* with a few modifications after the pilot. One simulation-trained faculty was present and acted both in the role of bedside nurse, as well as operated the SimMon monitor and stimuli. The initial vital signs were adjusted to remove the hypoxia and have an initial oxygen saturation of 97% to decrease the possible signal for pulmonary embolism. Because the pilot case required cuing from nursing to the gastrointestinal bleed, likely due to the distraction of the LVAD image and audio alarm, the cue was scripted to a five-minute timepoint if the team had not already identified. The second case was run for five residents (two PGY4, two PGY3, one PGY2), and the third case was run for five residents (two PGY4, one PGY3, two PGY2) and one medical student.

The learner response to this series of *in situ* cases was overwhelmingly positive. The learners evaluated the simulation with a brief two-question post case feedback, the first on the educational impact of knowledge and second on change in clinical care. All of the 15 learners (100%) between the pilot and two additional *in situ* cases reported benefiting in knowledge by participating in a debriefing on a critical and low frequency LVAD complication of a suction event. Ninety-three percent reported that the experience with the case would impact their clinical care. Multiple residents commented on the immediate realism and stress incited by the audio file with the HeartMate II low-flow alarm. There was robust discussion regarding the management of arrhythmia, and a learning point for each group was the tolerance of arrhythmia in patients with LVAD due to the mechanical pump maintaining perfusion. None of the residents in the three *in situ* cases interpreted the ultrasound image as representing a suction event.

The final version of the case with the modifications of normal oxygen saturation and standard cueing by embedded simulated bedside nurse to lower GI bleeding was run during the Western SAEM SIMposium competition. Four residents from an alternate institution were the learners in the case. The residents met all critical actions, provided volume for resuscitation, and consulted the LVAD team. The debrief focused on considerations for anticoagulant reversal. No formal post-evaluation was done of the learners in the final case because it was part of the Western SAEM SIMposium competition.

This case can be successfully implemented in a low-sim resourced environment with a low-fidelity mannequin with no interactive machination or even a simulated patient; using the physical printout image of the HeartMate on the patient and the audio stimuli, learners have an immediate buy-in into the simulation. A single facilitator can effectively run the case by using a laptop with slides for each stimulus, and can use slides to show initial vital signs as well as the vital changes by state as the scenario progresses. A single facilitator ran the second two *in situ* cases and navigated the communication demands by alternating between roles which the facilitator established in the simulation pre-brief with the learners, specifically stating that the faculty would be the voice of patient, bedside nurse, and wife for additional history. The learners simply had to be specific in their direction of questions and tasks throughout the case.

## Supplementary Information



## Figures and Tables

**Table:** 

#1	Electrocardiogram with ventricular tachycardia (Vtach)
#2	Image of LVAD Display with low flow alarm
#3	Chest X-Ray
#4	Audio file for LVAD low flow alarm
#5	Image of drive line site
#6	POCUS transthoracic ECHO image
#7	POCUS inferior vena cava image
#8	Venous Blood Gas
#9	Lactate
#10	Complete Blood Count
#11	Basic Metabolic Panel
#12	Troponin
# 13	International Normalized Ratio (INR)

**Table:** 

**Vitals:**	Heart Rate (HR) 154	Blood Pressure (BP) Unable to obtain *(manual cuff and Doppler MAP of 55)*
	Respiratory Rate (RR) 24	Temperature (T) 36.0°C
	Oxygen Saturation (O_2_Sat) 97% on room air	

**Figure:**
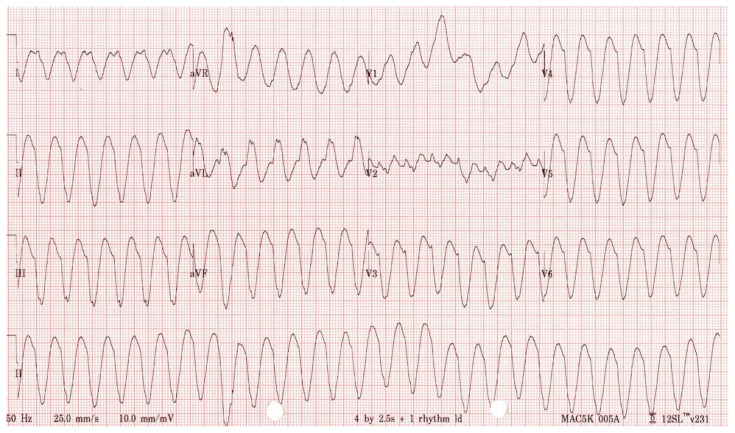


**Figure:**
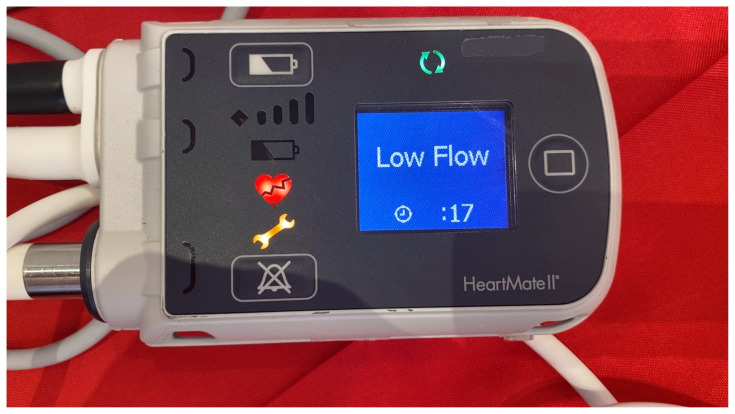


**Figure:**
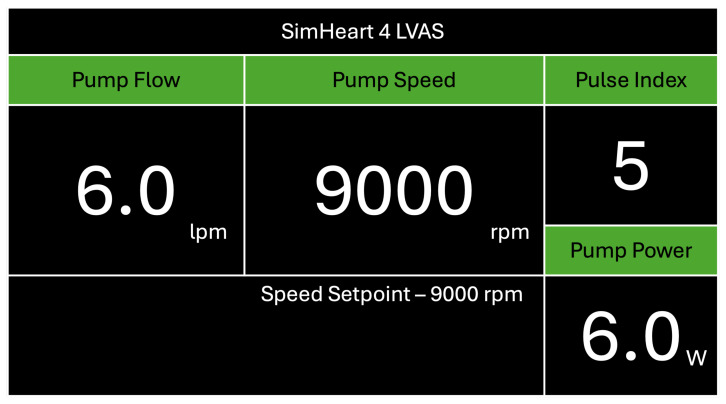


**Figure:**
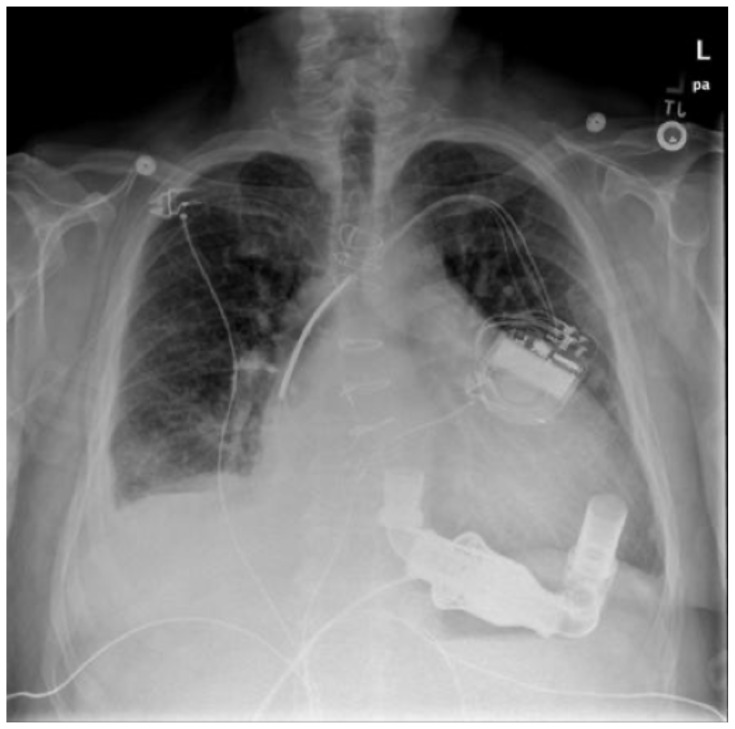


**Figure:**
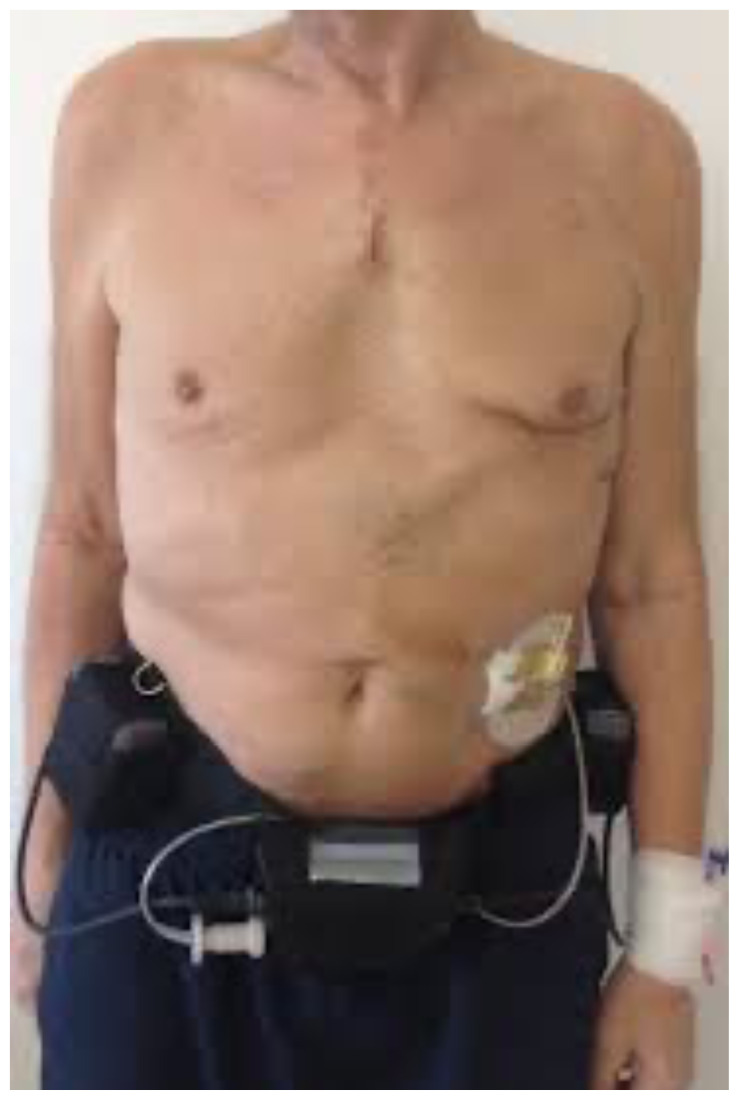


**Figure:**
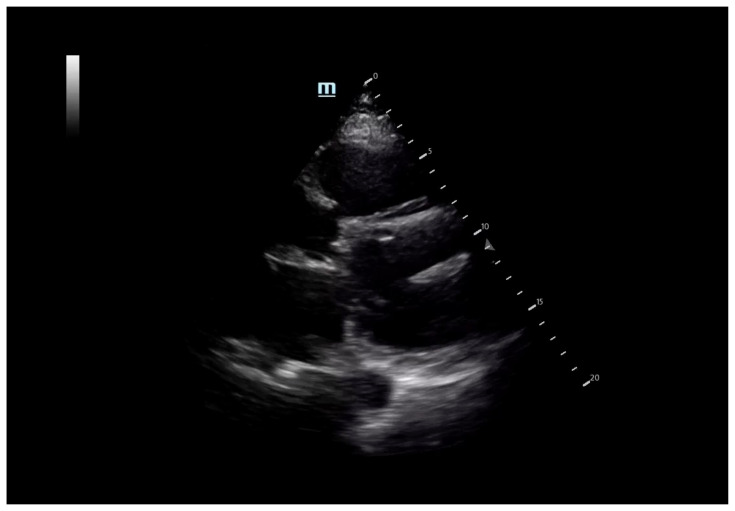


**Figure:**
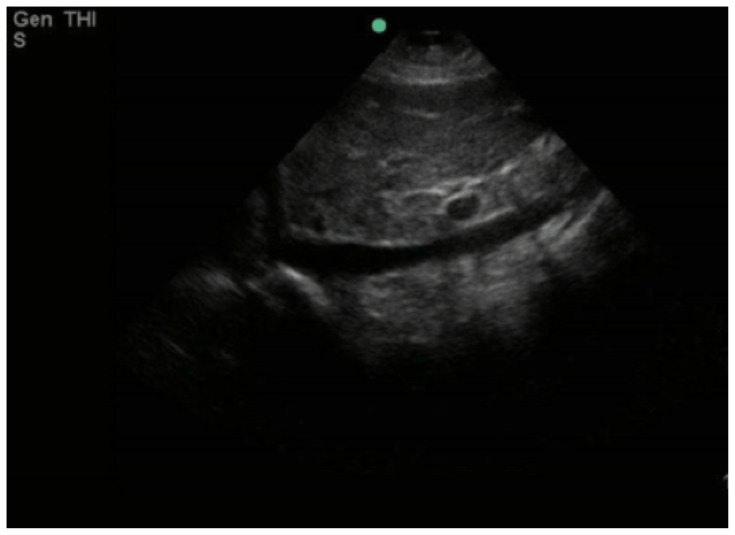


**Table:** 

*Venous blood gas (VBG)*	
pH	7.2
paCO2	62 mmHg
paO2	75 mmHg
HCO3	20 mmol/L
SaO2	85%

**Table:** 

*Lactic acid*	2.9 mmol/L

**Table:** 

*CBC (Complete Blood Count)*	
White blood count (WBC)	11.7 × 1000/mm^3^
Hemoglobin (Hgb)	7.3 g/dL
Hematocrit (HCT)	23%
Platelet (Plt)	360 × 1000/mm^3^

**Table:** 

*Basic metabolic panel (BMP)*	
Sodium	140 mEq/L
Potassium	4.1 mEq/L
Chloride	96 mEq/L
Bicarbonate (HCO3)	24 mEq/L
Blood Urea Nitrogen (BUN)	20 mg/dL
Creatinine (Cr)	1.1 mg/dL
Glucose	105 mg/dL

**Table:** 

*Troponin*	1.02 ng/mL (<0.1 ng/mL)

**Table:** 

*International Normalized Ratio (INR)*	2.56

**Table:** SIMULATION EVENTS TABLE:

Minute (state)	Participant action/trigger	Patient status (simulator response) & operator prompts	Monitor display (vital signs)
0:00 (Baseline)	Case begins. Patient is brought back by nurse and gives report to care team. Informs the team that blood pressure cannot be obtained.	Patient is able to give history regarding LVAD and bright red blood per rectum; he states, “I don’t feel well; I feel dizzy.” If physicians use manual blood pressure cuff and Doppler or stethoscope, a mean arterial pressure (MAP) of 55 will be provided on the monitor. Physicians ask for electrocardiogram to assess for rhythm given the patient’s elevated heart rate. HEARTMATE II initial output: Pump speed 9200 RPM Flow 2.5 L/min Power 4.0 Pulsatility Index (PI) 2.5 Confederates: Wife, Lizzy, is parking car. Nurse: not much experience with LVADs before, asks, “If there is no pulse, should we do chest compressions?!” She also does not know how to obtain MAP and looks for guidance from team.	T 36°C HR 154 BP —- RR 24 O2 97%
State 1 Evaluation	Physicians ask for EKG.	Electrocardiogram shows ventricular tachycardia. LVAD continues to alarm, says “suction event.” Nurse: “Should we shock him with an LVAD?”	HR 154 MAP 55
	Physicians order crystalloid or blood bolus for suction event. If no fluids or blood products ordered, go to State 2.	Patient remains arousable but not providing much history. LVAD has stopped alarming after receiving bolus. Exam: drive line site with no sign of infection. When fully exposed, large bloody bowel movement is identified. Wife enters: Can suggest use BP cuff and Doppler saying, “The doctors told us if you can’t get a blood pressure, you have to check for MAP if needed.” She is very anxious, provides additional history including description of bloody bowel movements if physicians have not successfully identified on exam.	MAP 55 HR 120 RR 24 O2 97%
	Consider differential for hypotension. Identify GI bleed.	If only one embedded simulation participant, nurse can cue for gastrointestinal bleed, “I think I saw blood under the patient.” Emergency blood products ordered.	MAP 55 HR 120 RR 24 O2 97%
State 2 Early Shock	No IVF or blood products given.	Patient MAP drops to 48 and O2 drops to 90%, and RN can suggest volume resuscitation to return to State 3.	MAP 48 HR 120 RR 24 O2 90%
State 3 Resuscitation	Emergent blood arrives, is being transfused. If learners reverse INR, move to State 4.	Labs result: Hgb 7 INR 2.56 CMP: Na 140, Cl 96, K 4.1 LVAD new output: Speed 9200 Flow 3.2 Power 4.2 PI 3.0 If team has already called LVAD team, they will call back at this point.	MAP 63 HR 110 RR 24 O2 95%
State Optimization	INR Reversal.	Patient’s HR improves to 105 and MAP improves to 65 with reversal of INR and slowing of GI bleeding causing hypovolemia.	MAP 65 HR 105 RR 22 O2 95%
Case Completion	Consult to LVAD/transfer to LVAD facility via Cardiac Care Unit transport.	Admission presentation completed; patient accepted. Case ends	

**Table:** 

**Critical Actions:** Team members measured blood pressure accurately in a patient with an LVAD by using a Doppler or placing an arterial line. The team recognized the low-flow alarm and developed a differential of the patient’s hypotension. The team obtained an electrocardiogram to determine the patient’s arrhythmia, and an ultrasound to diagnose a suction event. Initiated treatment with fluids. The team identified the source of hypovolemia as gastrointestinal hemorrhage and treated with blood products.	0:00

**Table:** 

	Milestone	Did not achieve level 1	Level 1	Level 2	Level 3
1	**Emergency Stabilization (PC1)**	□ Did not achieve Level 1	□ Recognizes abnormal vital signs	□ Recognizes an unstable patient, requiring intervention Performs primary assessment Discerns data to formulate a diagnostic impression/plan	□ Manages and prioritizes critical actions in a critically ill patient Reassesses after implementing a stabilizing intervention
2	**Performance of focused history and physical (PC2)**	□ Did not achieve Level 1	□ Performs a reliable, comprehensive history and physical exam	□ Performs and communicates a focused history and physical exam based on chief complaint and urgent issues	□ Prioritizes essential components of history and physical exam given dynamic circumstances
3	**Diagnostic studies (PC3)**	□ Did not achieve Level 1	□ Determines the necessity of diagnostic studies	□ Orders appropriate diagnostic studies. Performs appropriate bedside diagnostic studies/procedures	□ Prioritizes essential testing Interprets results of diagnostic studies Reviews risks, benefits, contraindications, and alternatives to a diagnostic study or procedure
4	**Diagnosis (PC4)**	□ Did not achieve Level 1	□ Considers a list of potential diagnoses	□ Considers an appropriate list of potential diagnosis May or may not make correct diagnosis	□ Makes the appropriate diagnosis Considers other potential diagnoses, avoiding premature closure
5	**Pharmacotherapy (PC5)**	□ Did not achieve Level 1	□ Asks patient for drug allergies	□ Selects an medication for therapeutic intervention, consider potential adverse effects	□ Selects the most appropriate medication and understands mechanism of action, effect, and potential side effects Considers and recognizes drug-drug interactions
6	**Observation and reassessment (PC6)**	□ Did not achieve Level 1	□ Reevaluates patient at least one time during case	□ Reevaluates patient after most therapeutic interventions	□ Consistently evaluates the effectiveness of therapies at appropriate intervals
7	**Disposition (PC7)**	□ Did not achieve Level 1	□ Appropriately selects whether to admit or discharge the patient	□ Appropriately selects whether to admit or discharge Involves the expertise of some of the appropriate specialists	□ Educates the patient appropriately about their disposition Assigns patient to an appropriate level of care (ICU/Tele/Floor) Involves expertise of all appropriate specialists
9	**General Approach to Procedures (PC9)**	□ Did not achieve Level 1	□ Identifies pertinent anatomy and physiology for a procedure Uses appropriate Universal Precautions	□ Obtains informed consent Knows indications, contraindications, anatomic landmarks, equipment, anesthetic and procedural technique, and potential complications for common ED procedures	□ Determines a back-up strategy if initial attempts are unsuccessful Correctly interprets results of diagnostic procedure
20	**Professional Values (PROF1)**	□ Did not achieve Level 1	□ Demonstrates caring, honest behavior	□ Exhibits compassion, respect, sensitivity and responsiveness	□ Develops alternative care plans when patients’ personal beliefs and decisions preclude standard care
22	**Patient centered communication (ICS1)**	□ Did not achieve level 1	□ Establishes rapport and demonstrates empathy to patient (and family) Listens effectively	□ Elicits patient’s reason for seeking health care	□ Manages patient expectations in a manner that minimizes potential for stress, conflict, and misunderstanding. Effectively communicates with vulnerable populations, (at risk patients and families)
23	**Team management (ICS2)**	□ Did not achieve level 1	□ Recognizes other members of the patient care team during case (nurse, techs)	□ Communicates pertinent information to other healthcare colleagues	□ Communicates a clear, succinct, and appropriate handoff with specialists and other colleagues Communicates effectively with ancillary staff

## INSTRUCTOR MATERIALS

**Case Title:** My Broken Heart

**Case Description & Diagnosis (short synopsis):** Fifty-seven-year-old male with past medical history of diabetes, hypertension, and viral cardiomyopathy complicated by biventricular heart failure and a HeartMate 3 Left ventricular assist device (LVAD) placement two years prior who presents with dizziness to a community hospital at two AM. Patient approaches triage with chief complaint of dizziness and states, “My LVAD alarm is going off.” While talking to the triage nurse in the chair, he has a near syncopal event and is brought back rapidly by nurse (embedded simulation participant). The team finds the patient to be in ventricular tachycardia with a mean arterial pressure (MAP) in the 50s. Additionally, the patient’s LVAD is alarming, indicating a suction event. The patient is found to have a lower gastrointestinal bleed requiring a transfusion of blood products. The team will resuscitate the patient by obtaining access and rapidly transfusing crystalloid until blood is available. Once crystalloid or blood is initiated, the suction event resolves. The team is unable to reach the LVAD service immediately for guidance, but they do call back once the patient is stabilized.

**Equipment or Props Needed:**

- High fidelity simulation mannequin
- Monitor with telemetry leads
- Intravenous line (IV) + IV pole
- Nasal cannula
- Non-rebreather mask
- Mock medications in syringes clearly labeled “for simulation use only”: amiodarone, procainamide, labetalol, norepinephrine
- Crystalloid bolus
- Blood products (packed red blood cells will suffice)
- Patient gown
- Doppler
- Manual blood pressure cuff
- Optional: Arterial line and set up
- Optional: LVAD (cardiology departments may have trainer devices; otherwise, any sort of box-like device can be used with print-outs or media images of LVAD settings)
- Red dye or liquid or red fabric to simulate gastrointestinal bleed

**Actors needed:**

- Nurse: uncomfortable with LVAD patients
- Wife: not present initially, and then provides additional information later in the case

**Stimulus Inventory:**

**Background and brief information:** Prior to the onset of the case, a pre-brief will occur. The content of the pre-brief will include an introduction to the mannequin and purpose of the simulation exercise.

The setting is a community emergency department. It is two AM. The patient arrives by private vehicle and is roomed immediately because a blood pressure could not be obtained using the mechanical blood pressure machines in triage. The patient is brought back to the care area by a nurse (embedded simulation participant) who informs the team that the patient is here because his LVAD alarm has been going off and that a full set of vital signs could not be obtained in triage.

**Initial presentation:** The patient initially presents awake and conversant. He is able to provide past medical history including biventricular heart failure status post LVAD placement, anticoagulation status, and presenting symptoms of dizziness and GI bleed. He will be able to inform the care team that his LVAD alarm started ringing in the middle of the night and has not stopped, therefore prompting his presentation.

**How the scene unfolds:** The team will recognize the need to use a manual blood pressure cuff and Doppler to obtain a mean arterial pressure instead of traditional blood pressure cuff. If they do not do this, the wife confederate will suggest it from prior physician instruction. The patient will have a MAP in the 50s. The patient’s LVAD will continue to alarm with a “low flow” message. The team will obtain an EKG and find the patient to be in ventricular tachycardia (Vtach) at a rate of 150s. If participants attempt to defibrillate the patient, the Vtach will not resolve.

The patient will then state that he is not feeling well. He will become more hypotensive to a MAP of 50. A bedside echocardiogram will show the presence of a suction event. The team will resuscitate the patient by obtaining access and rapidly transfusing crystalloid until blood is available. They will give blood when it becomes available, after which the suction event and ventricular tachycardia will resolve. The MAP will improve to 60 and tachycardia will improve to 110. The patient will be transferred to a hospital with an LVAD team via CCU transport. If, after resuscitation, the participants attempt to admit the patient to the hospital, the patient’s wife, now at the bedside, will request transfer to an LVAD facility, as per the patient’s cardiologist.

**Critical actions:**

- *Critical Action 1:* Measure blood pressure accurately in a patient with an LVAD by using a Doppler or placing an arterial line.
- *Critical Action 2:* Develop a differential of hypotension and low-flow alarm in an LVAD patient.
- *Critical Action 3:* Manage an arrhythmia in an LVAD patient in the setting of an acute suction event with fluids.
- *Critical Action 4:* Identify the source of hypovolemia as GI hemorrhage and treat it with blood products.

**Case Title:** My Broken Heart

**Chief Complaint:** 57-year-old male with past medical history of left ventricular assist device (LVAD) here with dizziness and complains, “My LVAD alarm is going off.”

**General Appearance:** middle-aged gentleman, appears tired and mildly diaphoretic

**Primary Survey:**

- **Airway:** phonating in clear, full sentences
- **Breathing:** bilateral breath sounds
- **Circulation:** no pulse (continuous flow hum)

**History:**

- **History of present illness:** 57 yo M with diabetes, hypertension, viral cardiomyopathy complicated by biventricular heart failure and HeartMate 3 LVAD placement two years prior who presents with chief complaint of dizziness and states, “My LVAD alarm is going off.” He was sleeping and then awoke from sleep because he heard his LVAD alarm going off. He went to the bathroom before coming to the ED and saw blood in his stool.
- **Past medical history:** Diabetes, hypertension, viral cardiomyopathy complicated by biventricular heart failure and LVAD
- **Past surgical history:** LVAD placement
- **Patient’s medications:** Aspirin, warfarin, insulin, metformin, amlodipine, lisinopril
- **Allergies:** Penicillin
- **Social history:** Smoker, social alcohol use, no illicit drugs
- **Family history:** None relevant

**Secondary Survey/Physical Examination:**

- **General appearance:** middle-aged gentleman, appears tired and mildly diaphoretic
- **HEENT:** within normal limits
- **Neck:** within normal limits
- **Heart:** hum, no heart sounds
- **Lungs:** mildly tachypneic, clear lungs
- **Abdominal/GI:** mild tender to palpation diffusely in lower abdomen. Driveline site noted
- **Genitourinary/Rectal:** when shorts are removed, brisk bright red blood per rectum
- **Extremities:** no edema
- **Back:** within normal limits
- **Neuro:** responding to questions slowly, follows commands, otherwise no focal deficits
- **Skin:** clammy. Drive line site is clean, dry, intact without surrounding erythema, induration, or warmth
- **Lymph:** within normal limits
- **Psych:** within normal limits

*Electrocardiogram with Ventricular tachycardia (VTACH)*

Buttner R, Burns E. Monomorphic VT Example 1. In Life in the FastLane.

https://litfl.com/ventricular-tachycardia-monomorphic-ecg-library/. Published Mar 19, 2023. Accessed February 28, 2024. CC BY-NC-SA 3.0.

*Image of LVAD Display with low flow alarm to place on mannikin*

Author’s own image and video

*Sim HeartMate Display*

Author’s own graphic

*Chest Xray*

Sharma R. Left ventricular assist device. In: Radiopaedia. https://radiopaedia.org/articles/left-ventricular-assist-device. Last revised Jan 24, 2024. Accessed February 28, 2024. CC BY-NC-SA 3.0.

*Audio file for LVAD low flow alarm* (included in PowerPoint file)

*Image of drive line site*

Image of drive line site. Left ventricular assist device, Hannover. In Wikimedia Commons. https://commons.wikimedia.org/wiki/File:Left_ventricular_assist_device_hannover.PNG Accessed October 16, 2024. CC BY-SA-3.0.

*POCUS Transthoracic ECHO Image*

Guy M. LVAD Cardiac US Images. JETem. https://jetem.org/download/lvad-cardiac-ultrasound-images/ Published March 22, 2025. Accessed April 1, 2025. CC BY-NC-SA 4.0.

*POCUS Inferior Vena Cava Image*

Carroll D. Inferior Vena Cava Ultrasound. In: Radiopaedia.

https://radiopaedia.org/cases/inferior-vena-cava-ultrasound-2?lang=gb July 2, 2018. Accessed Feb 28, 2024. CC BY-NC-SA 3.0

## OPERATOR MATERIALS

**Diagnosis:**

Lower gastrointestinal bleed causing low flow state resulting in suction event and presenting as LVAD low flow alarm.

**Disposition:**

Admission to inpatient LVAD team, or if not at tertiary facility, transfer with blood products to LVAD receiving facility via Cardiac Care Unit transport

## DEBRIEFING AND EVALUATION PEARLS

### My Broken Heart: LVAD

- Left ventricular assist devices (LVADs) are the most common mechanical circulatory assist device and are comprised of a mechanical pump that serves as a bridge to recovery, or heart transplantation, or as destination therapy for those non-surgical patients with severe heart failure and cardiogenic shock.^1^ These devices have distinct challenges for assessment and management in the emergency department and are increasingly presenting to emergency physicians, with one study noting 41% of LVAD patients had ED encounters. 2 The initial LVADs produced pulsatile flow, though newer models have continuous flow comprised of an inlet and outlet ports and a rotating element to increase arterial flow and pressure. The rotating element can be either axial as in HeartMate or centrifugal as in the HeartWare HVAD System. 1
- Important definitions and LVAD parameters:
  - Pump Speed (RPM): revolutions per minute which are set by the heart failure physician and determine pump flow. Typically, the speed is 2200–2800 rpm (HeartWare VAD) and 8000–10000 rpm (HeartMate II VAD) 1,6
  - Flow: Device flow = Rotator speed/Pump inflow-Pump outflow. Typically, the flow is 4–6 L/min. 1,6 Low flow rate can be caused by decreased preload or increased afterload states. Decreased preload to the device can be seen in low intravascular volume status, right ventricular failure, tamponade, thrombus, inflow cannula obstruction, or kinking. High afterload situations can occur if there is obstruction of the outflow cannula or a hypertensive emergency.
  - Pump Power: Pump power refers to the energy, specifically the current and voltage of the motor, and is affected directly by pump speed and flow. Typically, it is 4–6 Watts. 1,6 When flow is obstructed, power is reduced, though power is increased and flow decreased if a thrombus forms on the rotor.
  - Pulsatility Index (PI): this is related to the flow pulse through the pump measured and averaged over a 15-second interval for the HeartMate specifically. Typically, it is 1–10. 1,6 The PI will be affected by volume status and cardiac contractility. When preload and contractility increase, PI will increase; PI will decrease with decreased blood volume and afterload or with an obstruction to either inflow or outflow cannula causing low flow and power.
- **Initial evaluation**: you can determine normal function of the LVAD by auscultating to hear a continuous machinery hum over the precordium of the patient. In addition, review the external controller for LVAD parameters, battery life, and any alarms. Hemodynamic evaluation in the LVAD patient is complicated by this continuous flow and decreased or often absent peripheral pulses which make standard non-invasive blood pressure measurement ineffective. The mean arterial pressure (MAP) as obtained with a manual cuff and Doppler ultrasound over the radial or brachial artery is the perfusion measurement in the LVAD patient. Alternatives of course include placing an arterial line in the critical patient. Shock is defined as a MAP less than 60mmHg. 1,6
- **Suction events**: A suction event can occur if pump filling (ie, preload) is reduced, increasing negative pressure in the left ventricle (LV). This negative pressure sucks the LV wall over, covering the pump’s inlet cannula. The pump will alarm and display the low flow state, and speed will decrease to attempt to release the suction. This can trigger ventricular arrhythmias; management involves decreasing RPMs and increasing intravascular volume with volume resuscitation. 1
- **Arrhythmia management:** Atrial, ventricular, and both atrial and ventricular arrhythmias can be seen in the LVAD patient. Ventricular arrhythmias are reported in 22–59% of LVAD patients. Given that the patients with an LVAD maintain the cardiac output via the device, most patients can tolerate ventricular arrhythmias. However, ventricular arrhythmias can lead to inadequate LVAD flow and RV failure, and patients may often have automated implantable cardioverter defibrillators (AICDs) in place. In the case of a suction event, hemodynamic management is the best approach to manage ventricular arrhythmias. Cardioversion is indicated if the patient is unstable and in a clear shockable rhythm, though if the underlying cause is not addressed the arrhythmia may persist. 1
- **GI bleeding in LVAD**: Gastrointestinal bleeding is common with an incidence of 30% due to a number of factors such as anticoagulation/anti-thrombotics, thrombocytopenia/platelet dysfunction (acquired von Willebrand), and arteriovenous malformations (AVMs, thought to arise from the absent pulsatile flow). Hemodynamic instability and hemorrhagic shock can result from GI bleeding. Anti-thrombotics are key in the outpatient management of the LVAD patient to ensure adequate flow, with aspirin and warfarin as the typical anticoagulants with a target INR of 2.0–3.0, though higher for those patients with prior deep venous thrombosis, pulmonary embolism, inherited thrombophilia (INR goal 2.5–3.5) or those with atrial fibrillation or history of stroke (INR goal 2.5–3.0). In patients presenting with severe bleeding. 3
- **Anticoagulation reversal:** For the LVAD patient, the decision to reverse anticoagulation with administration of Vitamin K or fresh frozen plasma should be made in conjunction with the LVAD team because the risks of reversing anticoagulation could prevent LVAD function. The mainstay of treatment is to replete intravascular volume to address the decreased preload prompting the suction event with transfusion to match acute gastrointestinal blood losses and provide time for interventions to treat the cause of gastrointestinal bleed with either endoscopy, colonoscopy, or Interventional Radiology. 3
- **Disposition**: If not in a tertiary care setting, discuss with learners the appropriate transport indicated for a critical cardiac patient and how that would impact disposition at individual institutions.

**Other debriefing points:**

We found on some of the initial *in situ* pilots that the bedside US image could be confused with right heart strain, and residents with the original lower O2 saturation went down a pulmonary embolus pathway for diagnosis.

## SIMULATION ASSESSMENT

*My Broken Heart*

Learner: _________________________________________

***Assessment Timeline***

This timeline is to help observers assess their learners. It allows observer to make notes on when learners performed various tasks, which can help guide debriefing discussion.

**Critical Actions:**

□ Team members measured blood pressure accurately in a patient with an LVAD by using a Doppler or placing an arterial line.
□ The team recognized the low-flow alarm and developed a differential of the patient’s hypotension.
□ The team obtained an electrocardiogram to determine the patient’s arrhythmia, and an ultrasound to diagnose a suction event. Initiated treatment with fluids.
□ The team identified the source of hypovolemia as gastrointestinal hemorrhage and treated with blood products.

**Summative and formative comments:**

**Milestones assessment:**
