# Supplementary material for: My Broken Heart
Source: J Educ Teach Emerg Med. 2025 Apr 30;10(2):S1–S30. doi: 10.21980/J85W7R (PMC12054096; doi:10.21980/J85W7R)
Supplement: Supplementary file 1 [file 10-2-S1-supp1.pptx]

## Slide 1
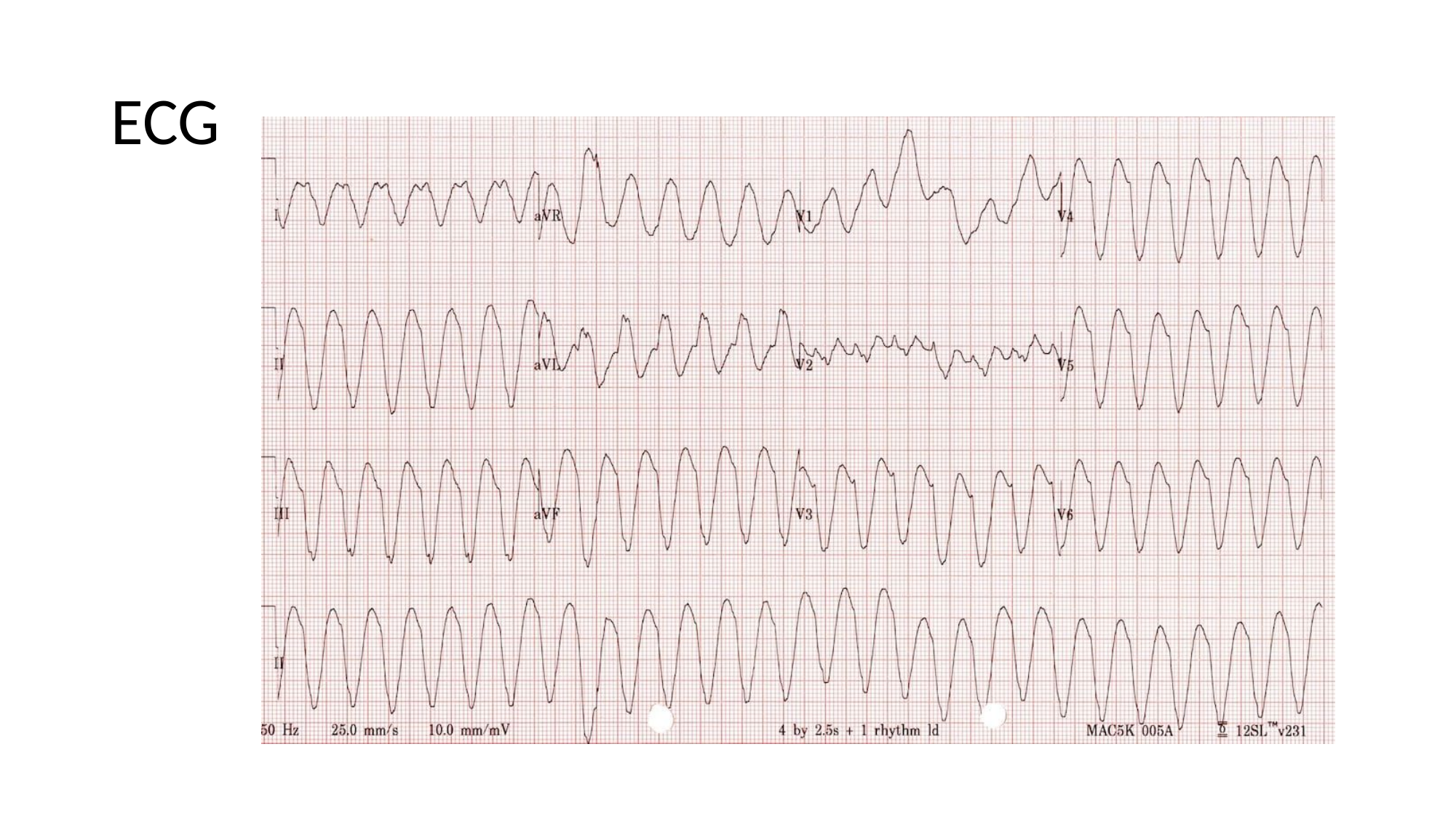

# ECG

## Slide 2
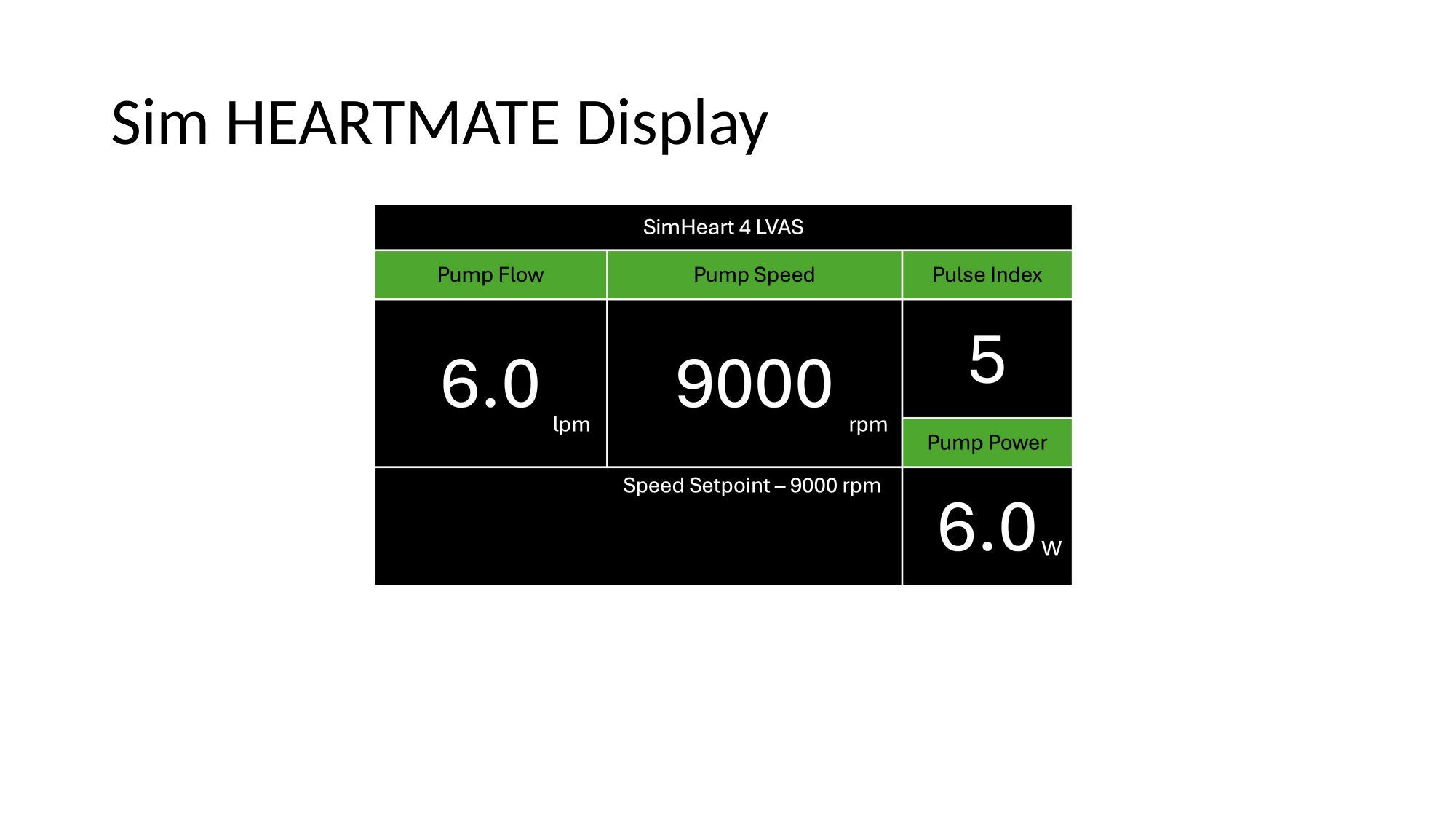

# Sim HEARTMATE Display

## Slide 3
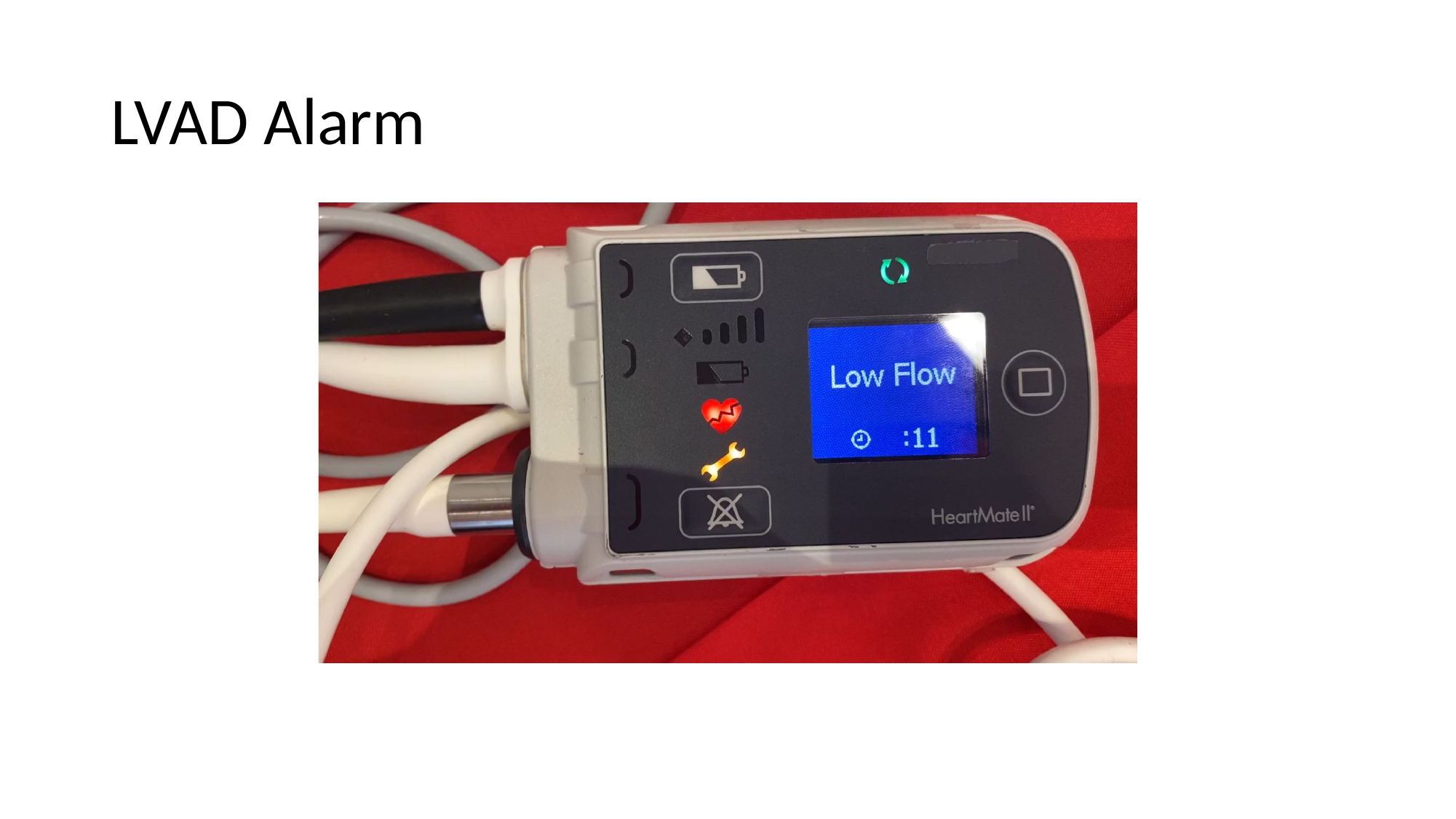

# LVAD Alarm

## Slide 4
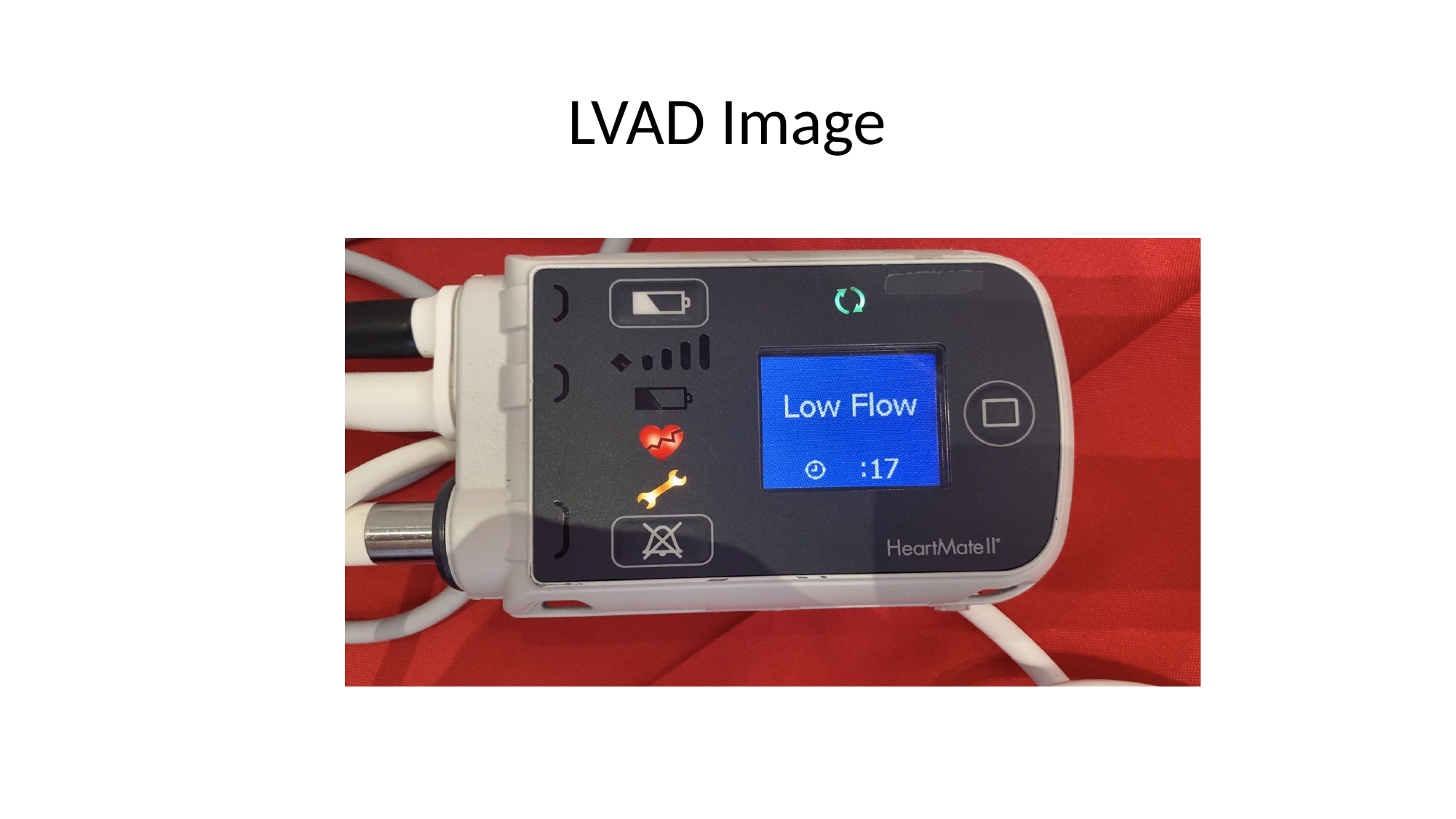

# LVAD Image

## Slide 5
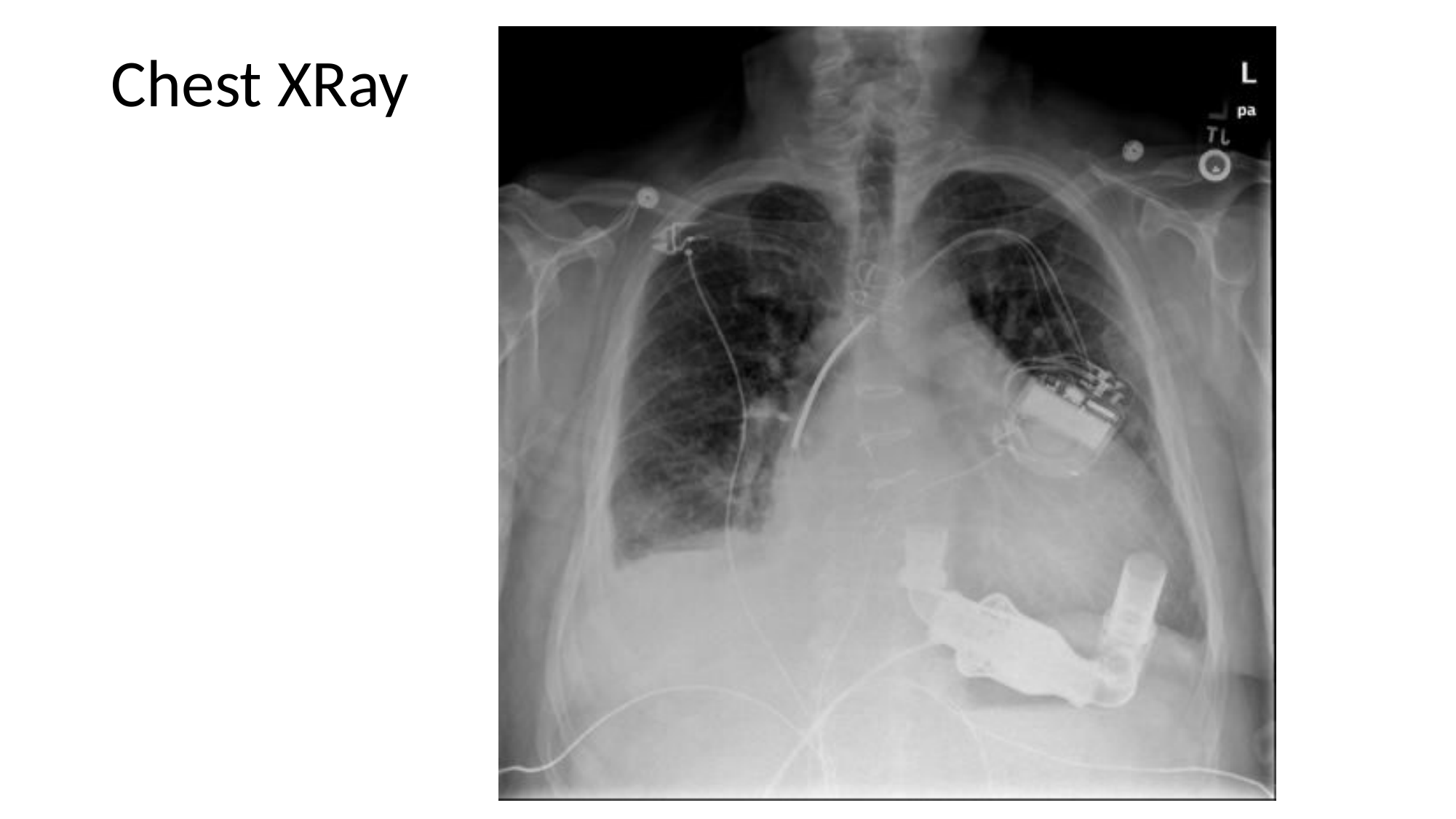

Chest XRay

## Slide 6
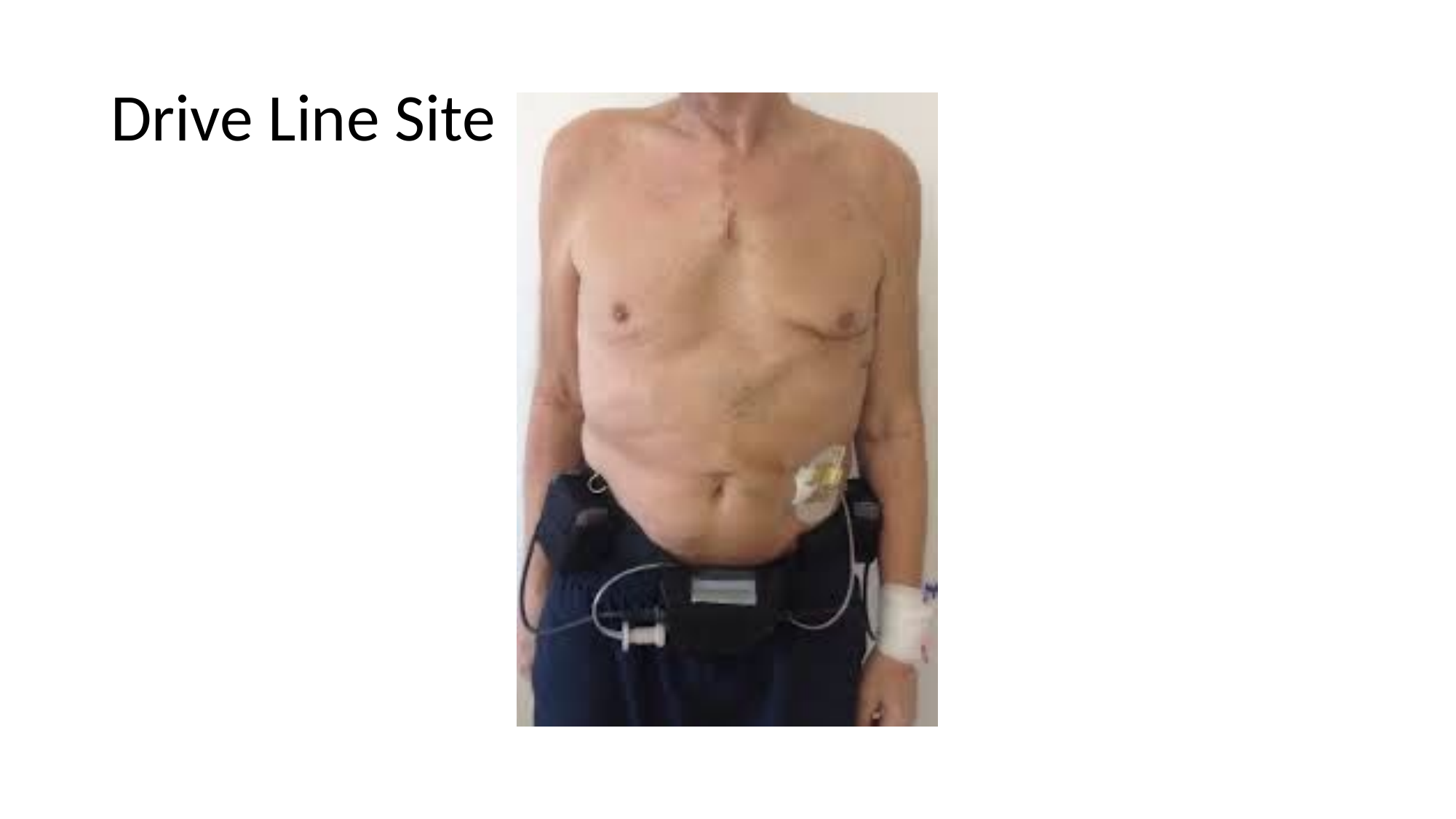

# Drive Line Site

## Slide 7
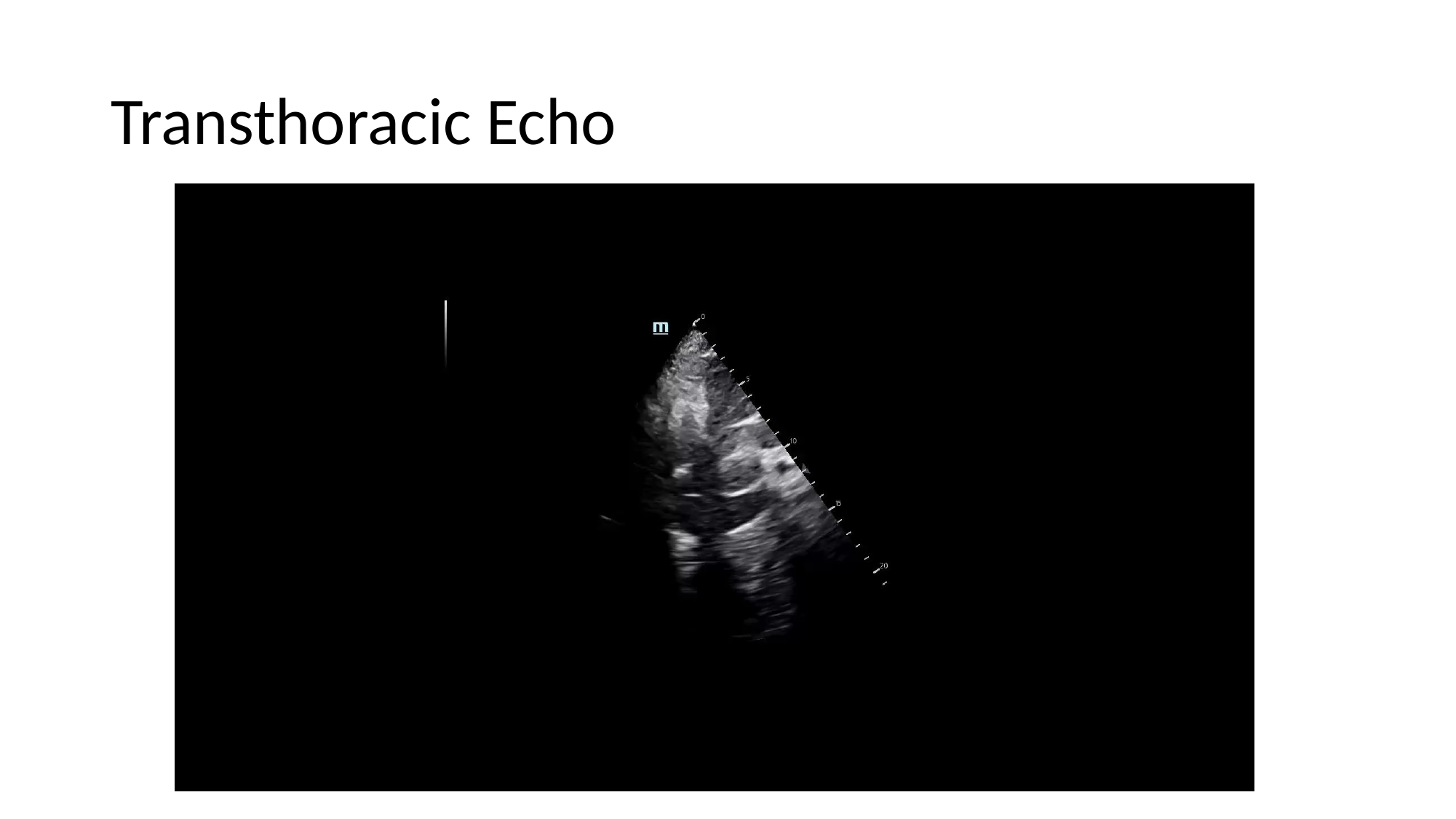

# Transthoracic Echo

## Slide 8
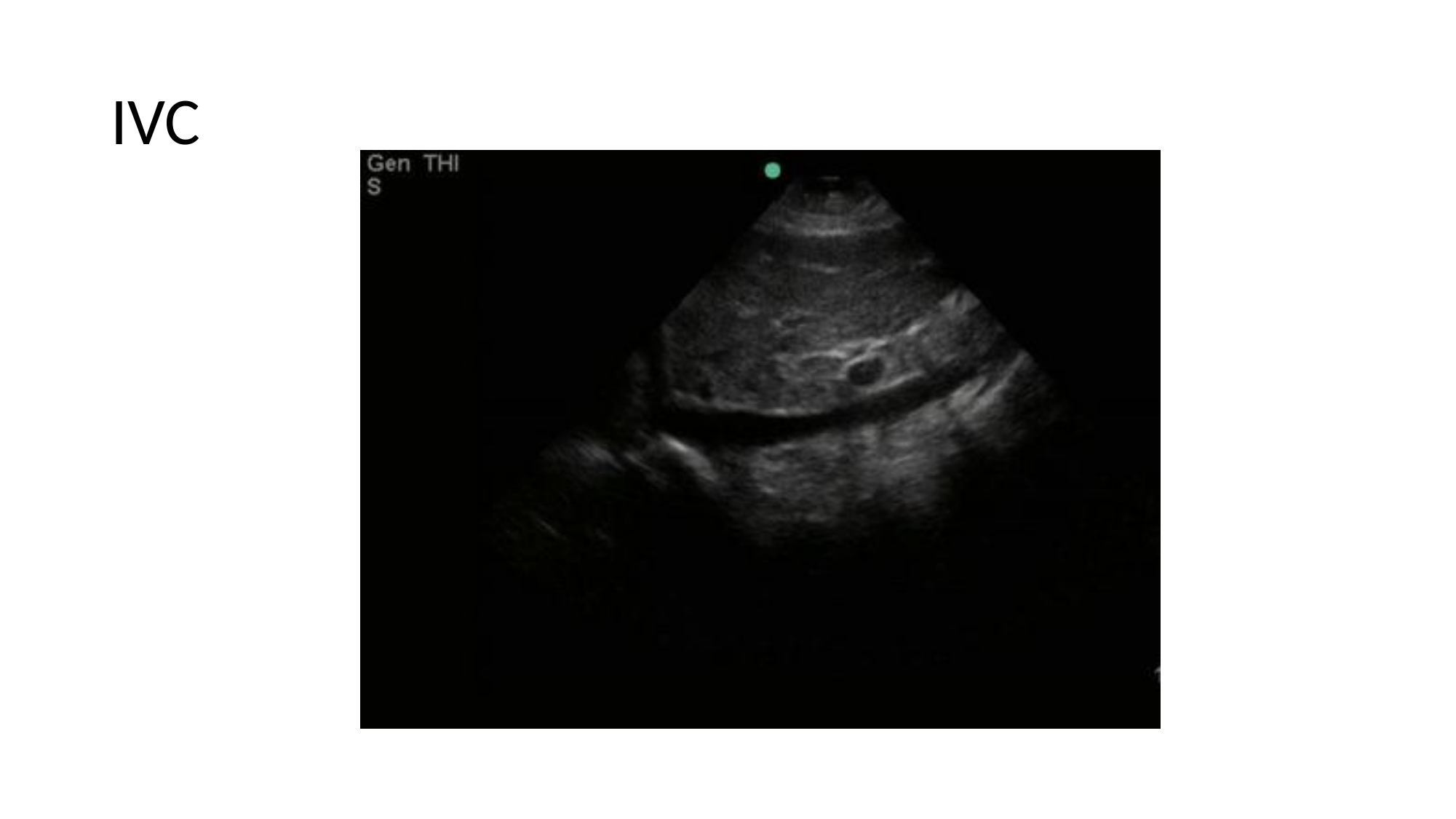

# IVC

## Slide 9
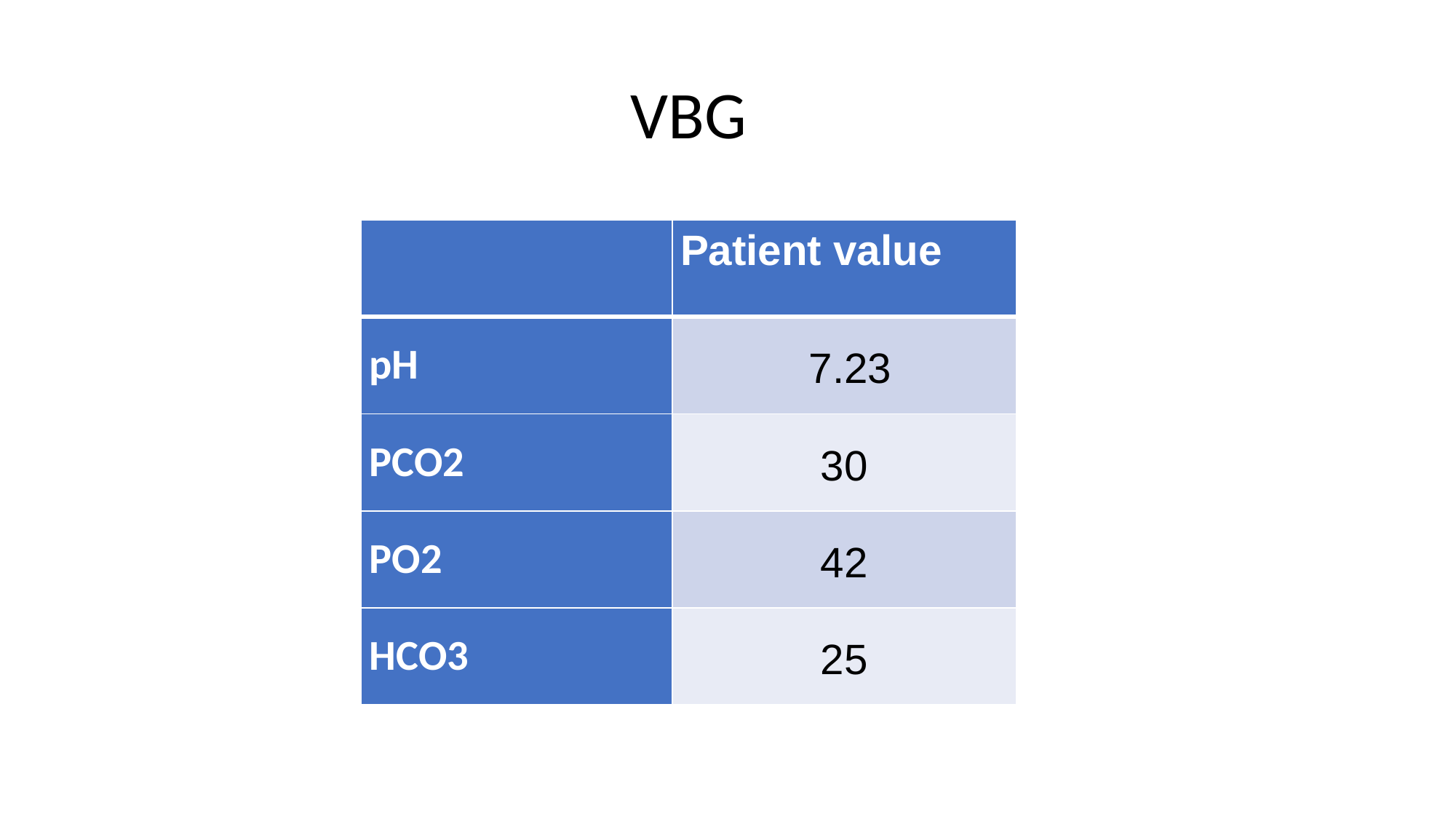

# VBG
| | Patient value |
| --- | --- |
| pH | 7.23 |
| PCO2 | 30 |
| PO2 | 42 |
| HCO3 | 25 |

## Slide 10
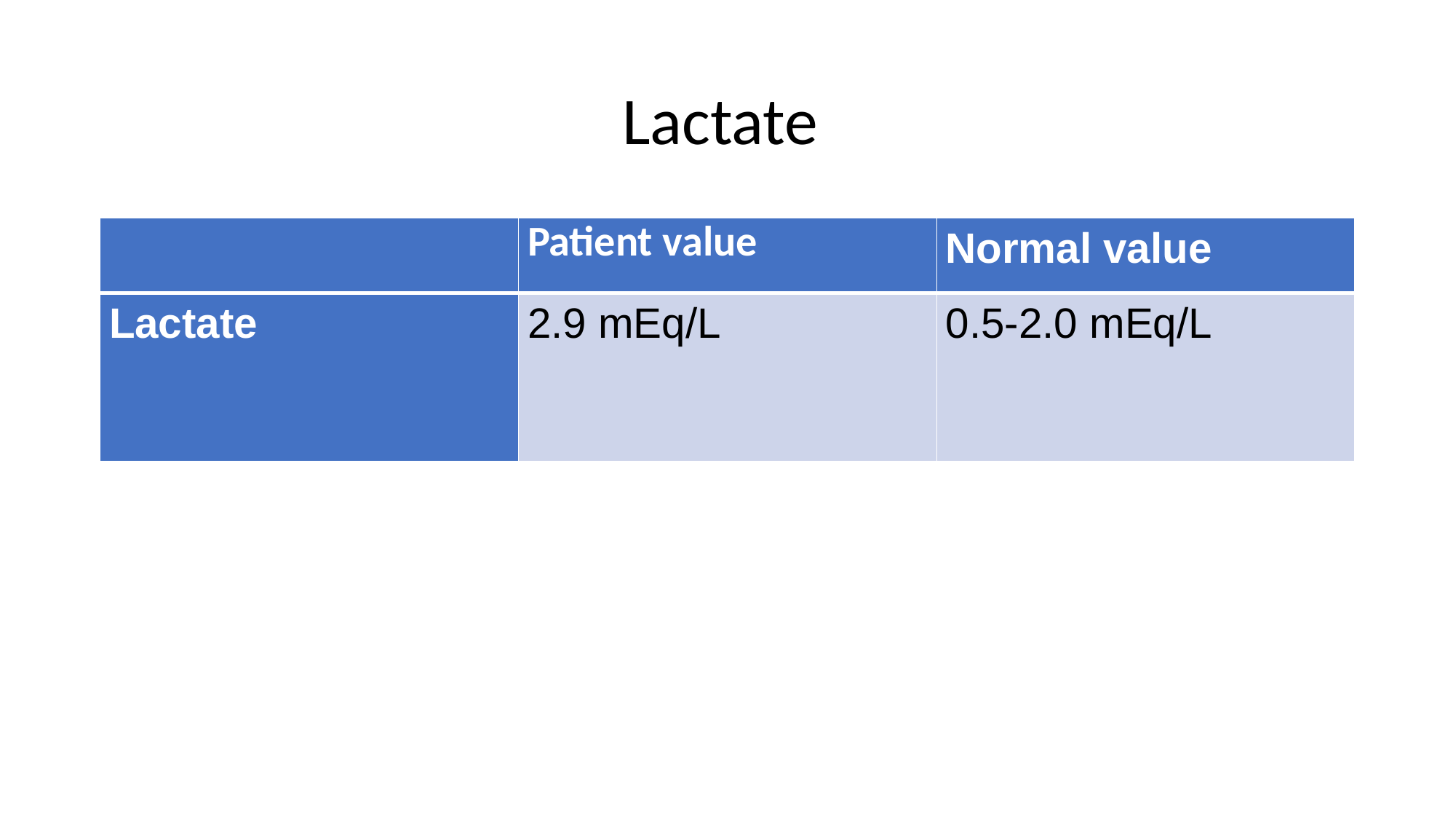

# Lactate
| | Patient value | Normal value |
| --- | --- | --- |
| Lactate | 2.9 mEq/L | 0.5-2.0 mEq/L |

## Slide 11
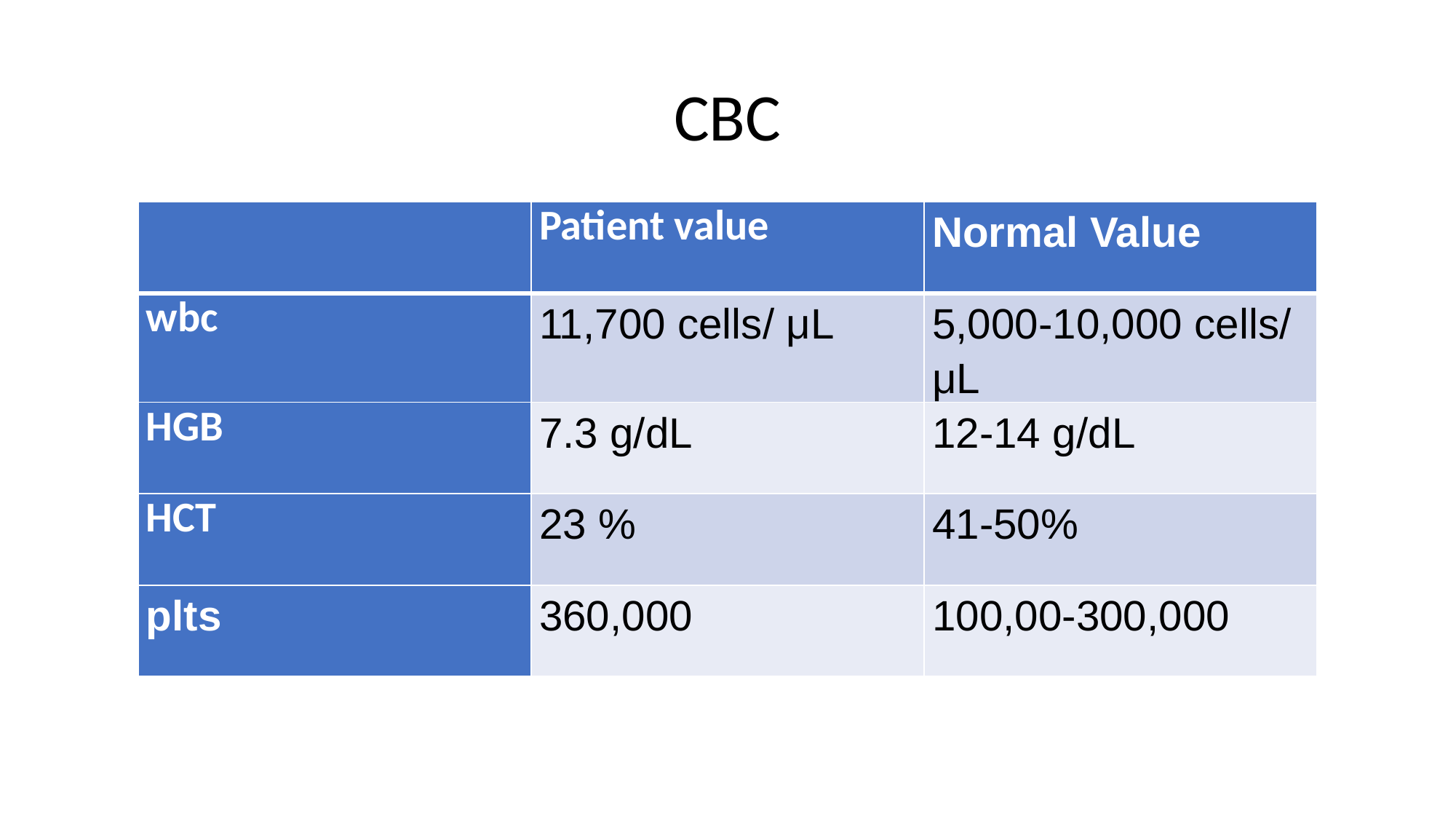

# CBC
| | Patient value | Normal Value |
| --- | --- | --- |
| wbc | 11,700 cells/ μL | 5,000-10,000 cells/ μL |
| HGB | 7.3 g/dL | 12-14 g/dL |
| HCT | 23 % | 41-50% |
| plts | 360,000 | 100,00-300,000 |

## Slide 12
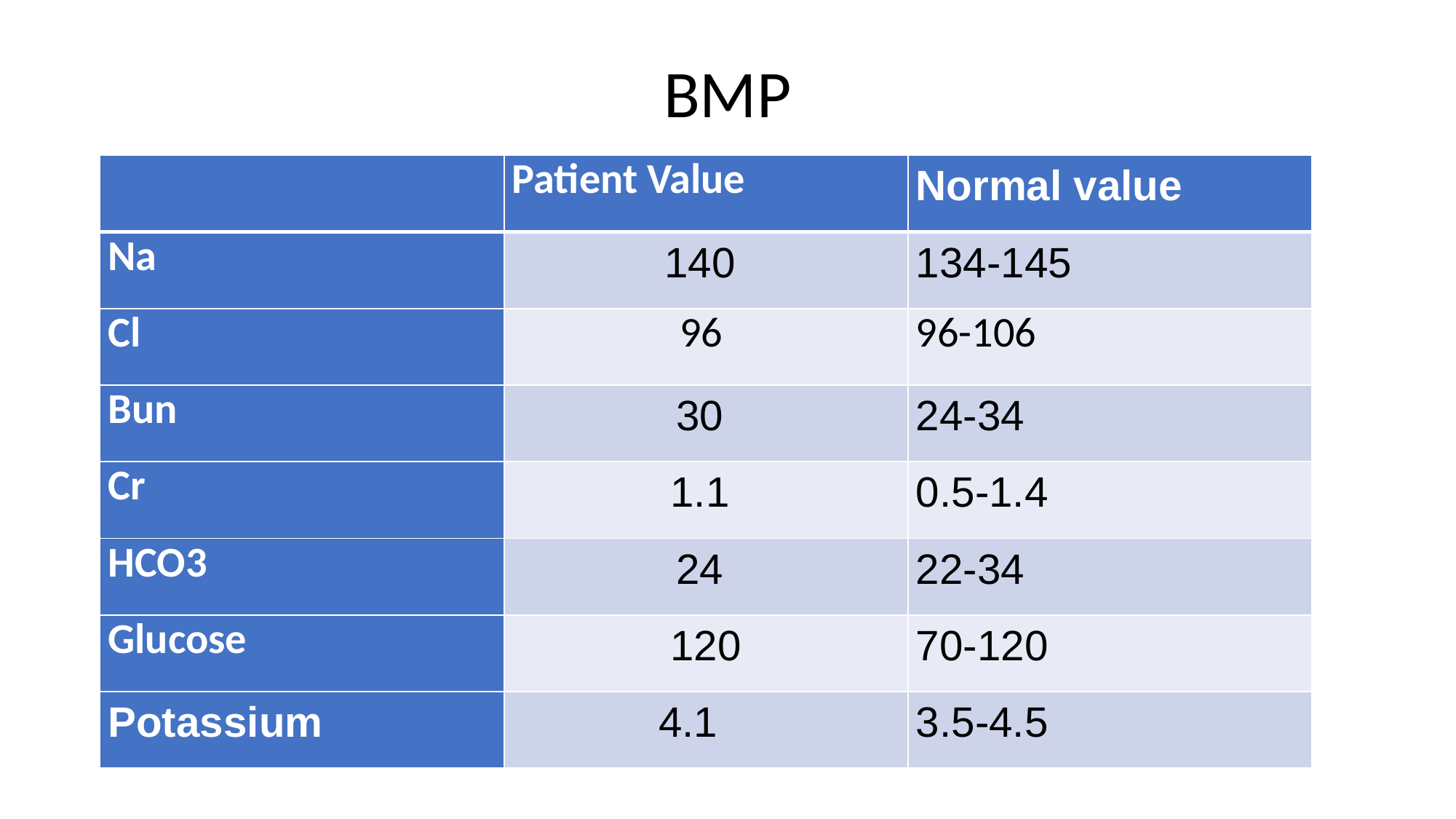

# BMP
| | Patient Value | Normal value |
| --- | --- | --- |
| Na | 140 | 134-145 |
| Cl | 96 | 96-106 |
| Bun | 30 | 24-34 |
| Cr | 1.1 | 0.5-1.4 |
| HCO3 | 24 | 22-34 |
| Glucose | 120 | 70-120 |
| Potassium | 4.1 | 3.5-4.5 |

## Slide 13
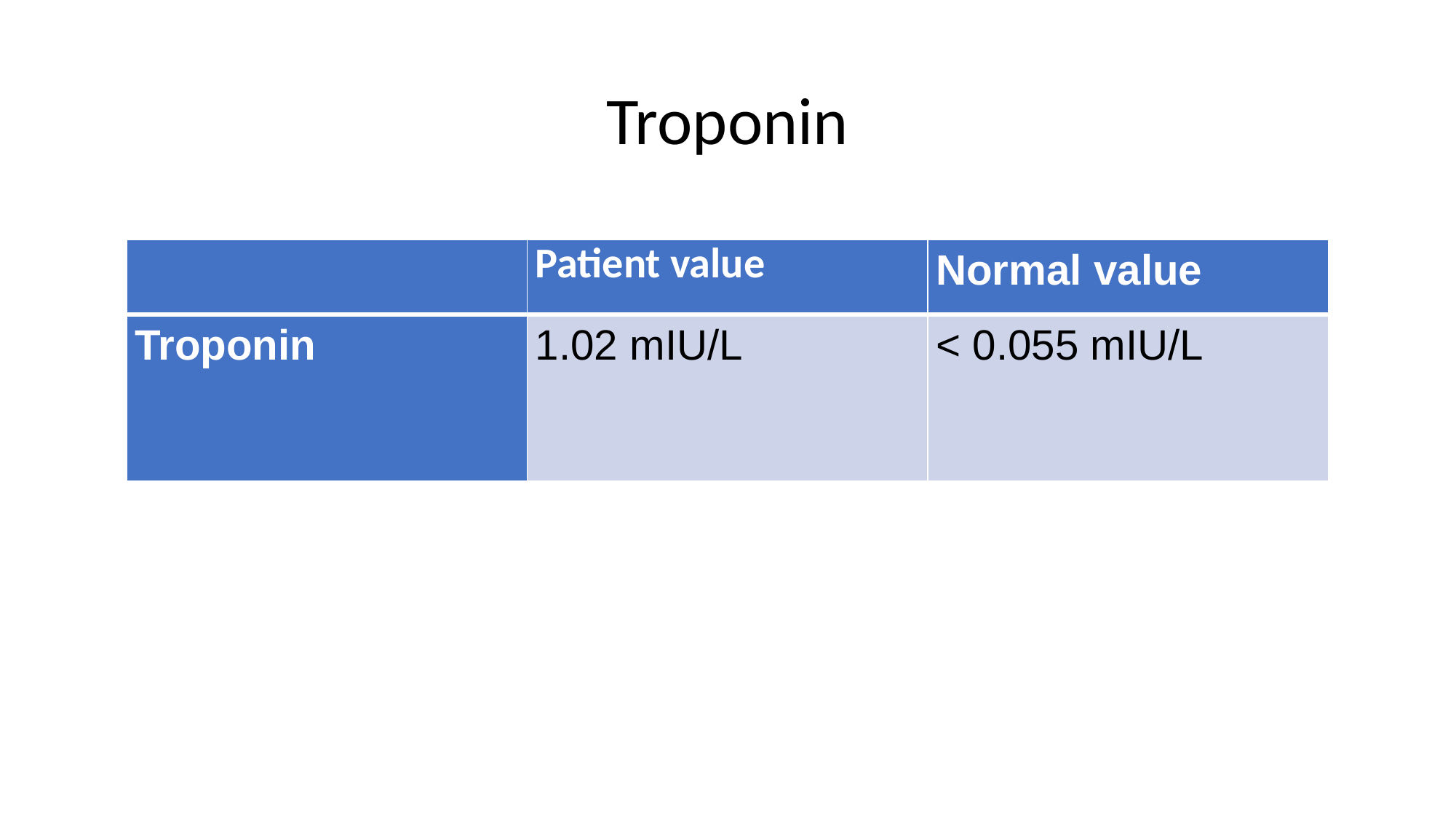

# Troponin
| | Patient value | Normal value |
| --- | --- | --- |
| Troponin | 1.02 mIU/L | < 0.055 mIU/L |

## Slide 14
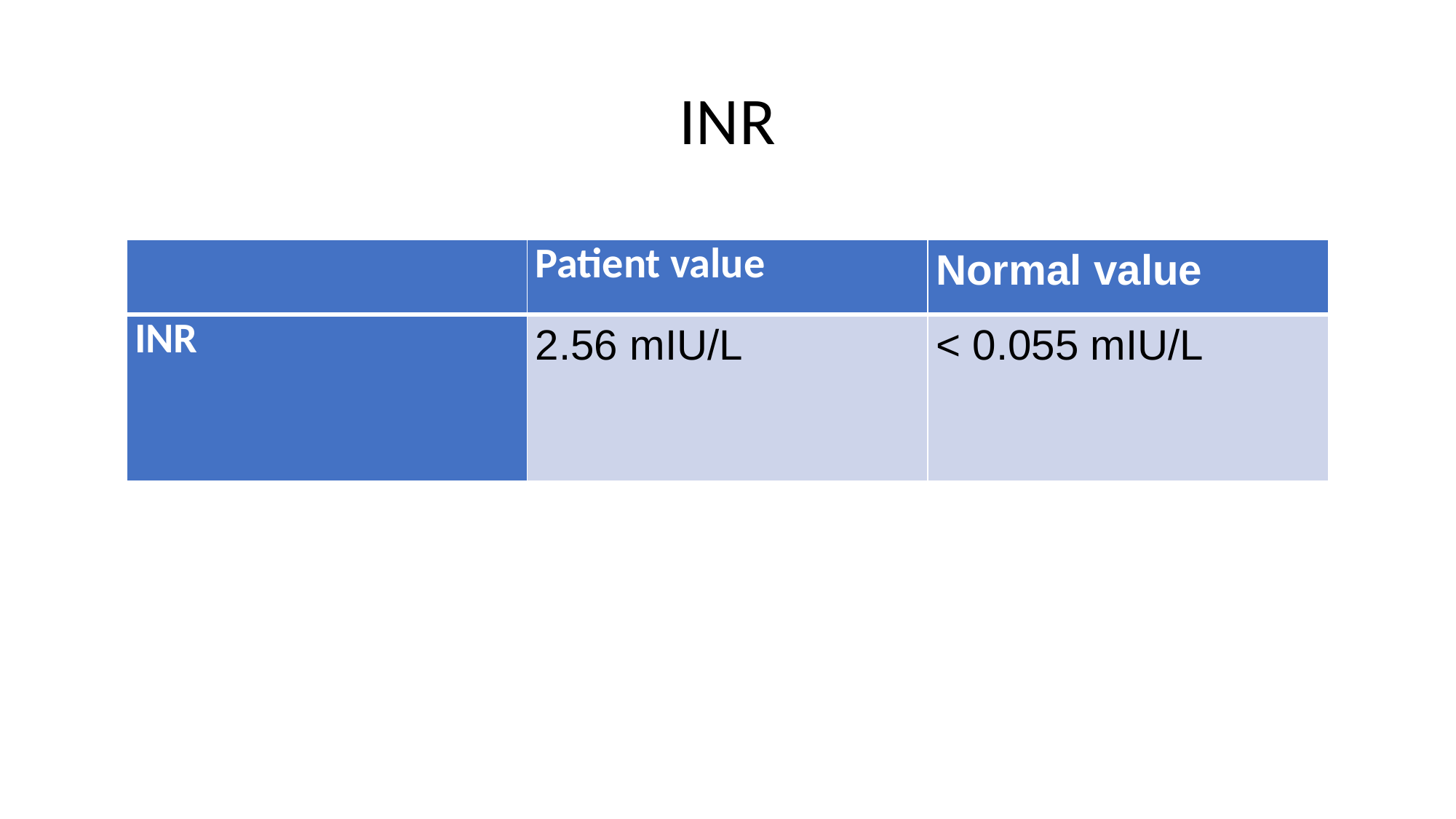

# INR
| | Patient value | Normal value |
| --- | --- | --- |
| INR | 2.56 mIU/L | < 0.055 mIU/L |
